# A Role for Estrogen Receptor Phosphorylation in the Resistance to Tamoxifen

**DOI:** 10.4061/2011/232435

**Published:** 2011-07-12

**Authors:** Renée de Leeuw, Jacques Neefjes, Rob Michalides

**Affiliations:** Department of Cell Biology, The Netherlands Cancer Institute, Plesmanlaan 121, 1066CX Amsterdam, The Netherlands

## Abstract

About two thirds of all human breast cancer cases are estrogen receptor positive. The drug of first choice for these patients is tamoxifen. However, about half of the recurrences after removal of the primary tumor are or become resistant to this drug. While many mechanisms have been identified for tamoxifen resistance in the lab, at present only a few have been translated to the clinic. This paper highlights the role in tamoxifen resistance of phosphorylation by different kinases on different sites of the estrogen receptor. We will discuss the molecular pathways and kinases that are involved in phosphorylation of ER*α* and how these affect tamoxifen resistance. Finally, we will elaborate on the clinical translation of these observations and the possibility to predict tamoxifen responses in patient tumor samples before treatment onset. The findings made originally on the bench may translate into a better and personalized treatment of breast cancer patients using an old and safe anticancer drug: tamoxifen.

## 1. Introduction

Worldwide, some 1.5 million women are diagnosed with breast cancer annually. Approximately, 70% of human breast cancer expresses estrogen receptor alpha (ER*α*). These tumors are eligible to endocrine therapy. Over the last 30 years, tamoxifen has been the antiestrogen of first choice. However, about half of the recurrences in ER-positive breast cancer do not respond to tamoxifen, which is due to either acquired resistance or to intrinsic insensitivity to tamoxifen [1, 2]. From experimental studies, many different mechanisms have been suggested to explain resistance, including activation of kinase pathways or inactivation of pRb, that render the tumor cell independent of the ER pathway for its proliferation [2]. However, with exception of cErbB2 (neu) overexpression, which mostly, but not exclusively occurs in ER*α*-negative breast cancer [3, 4], currently none of the resistance mechanisms identified have been translated into clinical implementation.

 It is evident that multiple factors are involved in tamoxifen resistance. Therefore, they should be examined together as an integrated set of predictive markers for diagnosis of individual patients. Not only the number of clinically relevant indicators for tamoxifen resistance is unknown, but also the proportion in which a particular marker contributes to resistance in patients is unclear. The relative contribution of these factors should be defined and potentially integrated into a combined set of predictive markers for tamoxifen responses of individual patients. 

Tamoxifen stimulates the growth of osteoblasts, while it inhibits ER*α*-positive breast tumor cells. These two opposing effects of tamoxifen on cell growth can be explained by the fact that tamoxifen is a partial antagonist, acting as an agonist under particular conditions [5]. Tamoxifen resistance is usually due to a direct effect on ER*α*; tamoxifen may acquire agonistic properties for transactivation of ER*α* [6]. Therefore, a molecular understanding of the underlying mechanism of tamoxifen resistance could result in markers that specify how patients will respond to endocrine therapy. The potential translation of these markers into clinical evaluation has to be examined with historical material and ultimately in a prospective study. Identification of markers predicting the antibreast cancer response to tamoxifen would have major clinical implications. Currently, the clinical benefit of tamoxifen is similar to that of aromatase inhibitors, although the side effects of the drugs markedly differ. Ultimately, by finding predictive markers, responsiveness to tamoxifen can be defined before treatment and patients will only receive tamoxifen if they are likely to benefit from it. And in case of resistance, patients may still respond to another treatment modality, such as aromatase inhibitors or the full antiestrogen antagonist, like fulvestrant, which may still be beneficial [5].

The estrogen receptor superfamily consists of two homologous nuclear receptors: ER*α* and ER*β*. ER*β* is encoded by a different gene, and the two receptors exhibit different transcriptional activities and functions in breast cancer [7]. Because the phosphorylation of ER*β* and a potential role in tamoxifen resistance have not been well characterised, we will not discuss this estrogen receptor subtype.

In this chapter, we focus on phosphomodifications of ER*α* in tumor cells that, by themselves, do not affect the female hormone estradiol (E2) dependency of the tumor cells for proliferation, but could affect the response to tamoxifen. We will address the following questions: which phosphorylation sites are identified on ER*α*? How do these sites become phosphorylated? Which sites are associated with tamoxifen resistance? How does tamoxifen sensitivity become affected without any effect on E2 dependency? Which molecular pathways upstream or downstream of the phosphorylated ER are involved in this form of tamoxifen resistance? Which clinical data are in support of tamoxifen resistance due to phosphorylated ER*α*?

## 2. Effects of Phosphorylation on the Structure of ER**α** Which Are Relevant for Tamoxifen Resistance

Phosphorylation of the estrogen receptor may change the 3-dimensional structure of the protein. Unfortunately, thus far no full-length ER*α* has been crystallised. This complicates characterisation of structural changes upon ligand binding or posttranslational modifications, such as phosphorylation. Furthermore, a conformational change due to phosphorylation could have consequences for the action of estrogens and antiestrogens. X-ray crystallography studies have thus far been performed on the ligand binding domain (LBD) of ER*α*. Estradiol binds to amino acids Glu353 from helix 3(H3), Arg394 from H5, and to His524 from H11 in the LBD of ER [8], whereas D351 in the LBD is critical for the interaction with the antiestrogen. Specific mutation of D351 into D351Y resulted in a receptor that shows an estrogenic, instead of an antiestrogenic, response to tamoxifen [9]. 

Coactivators have a common signature motif, LXXLL, with which they can interact with ER*α* in a hormone-dependent manner [10]. Whereas in a nonligand-bound state helix 12 is highly mobile, upon binding of an agonist it takes a more fixed position, stabilising the conformation of ER*α*. Helix 12 forms a charge clamp with helix 3, creating a hydrophobic groove to which a coactivator can bind. In contrast, crystallography shows that, when an antagonist, such as tamoxifen, binds to the LBD, helix 12 itself occupies the coactivator binding site, rendering ER*α* inactive [11–13]. Structural changes of ER can influence coregulator binding and hence potentially the response to ligands. 

Besides binding to the LBD in the AF-2 domain, coactivators also bind to the AF-1 domain of ER*α*, in a ligand-independent manner. Phosphorylation of sites within or outside the AF-1 region may affect the AF-1-dependent binding of cofactors as well. 

Phosphorylation of particular sites, especially of S118 and S305, affects the binding of coactivators in the presence of tamoxifen [14]. In case of S305, this is due to an altered conformation of ER*α*, which can be measured by fluorescence resonance energy transfer (FRET) [15]. In the presence of tamoxifen, S305 phosphorylation changes the orientation between ER*α* and coactivator SRC-1 [14]. This altered orientation renders ER*α* transcriptionally active in the presence of tamoxifen. An altered conformation of ER*α* due to phosphorylation of S305 resulted in a tamoxifen-resistant phenotype of ER*α*, not only measured by FRET, but also by biological assays [6, 16]. Not only tamoxifen but also arzoxifene is converted from an antagonist into an agonist after the S305 phosphorylation-induced conformational arrest of ER*α* [16]. These findings strongly suggest that subtle changes in the conformation of ER*α* upon binding to antiestrogens are at the basis of resistance to antiestrogens. This provides the framework to consider a role for phosphorylation of ER*α* in resistance to tamoxifen.

## 3. ER**α** Phosphorylation Sites with a Putative Role in Tamoxifen Resistance

**Figure 1:**
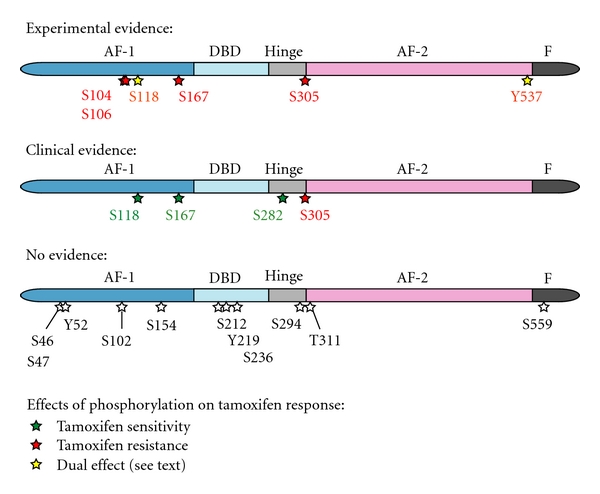
ER*α* phosphorylation involved in tamoxifen response. From left to right: AF-1 domain, DNA-binding domain (DBD), hinge region, AF-2 domain, and F domain containing helix 12.

Several kinase pathways have been associated with tamoxifen resistance, including activation of the protein kinase A (PKA) [17], mitogen-activated protein kinase (MAPK) [18] and p21-activated kinase-1 (PAK-1) signaling pathways [19]. These kinases induce phosphorylation of ER*α* or of its coregulators. This paper focuses on the phosphorylation sites on ER*α* that could contribute to an altered response to tamoxifen and on which kinase pathways and upstream activators are involved. A summary of the putative phosphorylation sites in ER*α* is presented in Figure 1 and Table 1. They are discussed separately below.

### 3.1. S102/S104/S106

Serine residues S102, S104, and S106 at the N-terminal AF-1 region of ER*α* are phosphorylated by glycogen synthase kinase-3 (GSK-3) and by extracellular signal-regulated kinases 1 and 2 (ERK1/2) and mitogen-activated protein kinase (MAPK) (=MEK1/2) pathways. These modifications lead to ligand-independent transcription of ER*α* and to an agonistic activity of tamoxifen [22, 23]. S102, a phosphorylation site discovered by mass spectrometry, requires concurrent phosphorylation of S104 [20]. ER*α* phosphorylation by GSK-3, which also targets S118, stabilizes ER*α* without ligand and modulates ER*α* transcriptional activity upon ligand binding. S104 and S106 can also be phosphorylated by the CDK2/cyclinA complex [24]. Cyclin A has been reported as a predictive marker for tamoxifen resistance in breast cancer patients [51].

### 3.2. S118

Serine 118 is one of the most reported phosphorylation sites of ER*α*. It is targeted by a number of kinase pathways: MAPK, GSK-3, IKK*α*, CDK7, and mTOR/p70S6K. S118 phosphorylation by MAPK increases binding of coactivator SRC3 [25] and renders ER*α* hypersensitive to estradiol [26]. Phosphorylated S118 decreases ER*α* affinity for tamoxifen and reduces binding to DNA, when ER*α* is tamoxifen bound [25]. In a tamoxifen-resistant cell line obtained by selection after prolonged exposure to tamoxifen, MAPK activity was found to be elevated and S118-P was increased [26]. Upstream, the RAS/MAPK pathway can be activated by IGF stimulation inducing phosphorylation of ER*α* S118 and resulting in ER*α* activation and enhanced response to estradiol [18]. Estradiol and EGF can induce the ERK1/2 MAPK pathway, which also leads to S118 phosphorylation of ER*α* [27]. Estrogen-dependent phosphorylation of S118-P can occur not only through the ERK1/2 MAPK pathway, but also by IKK*α* [28] and CDK7, a subunit of transcription factor II H [29]. 

In MCF7 cells, the receptor tyrosine kinase RET mediates ER*α* phosphorylation at S118 and S167 via the mTOR/p70S6K pathway [30]. Activation of RET leads to estrogen-independent transcriptional activation of ER-dependent genes and resistance to tamoxifen, strongly suggesting that RET activity acts through the estrogen receptor. This hypothesis is supported by a chromatin immunoprecipitation (ChIP) study on ER*α* and S118 mutants [52]. Phosphorylation of S118 influences the recruitment of coregulators to ER*α*-regulated genes pS2, c-myc, and cyclin D1 and affects E2-induced gene expression. The nonphosphorylatable S118A mutant has a greater impact on genes regulated through nonclassical mechanisms, such as ER*α* binding to fos/jun on an AP-1 promoter, than on estrogen responsive elements (ERE). 

The clinical relevance of S118 phosphorylation in tamoxifen resistance is still unresolved. On the one hand, S118-P has been associated with a more differentiated phenotype, good prognosis, and better response to tamoxifen [28], which is supported by other studies (see [31], Wigerup et al. unpublished data). Most importantly, these studies reported that the S118 phosphorylation had no effect on progression of disease or survival without tamoxifen treatment [31], thereby emphasizing that S118 phosphorylation is a clear predictive marker for response to tamoxifen in these studies.

On the other hand, S118 phosphorylation was negatively correlated with response to endocrine therapy in patients in other studies [32–34]. Nontreated patients have a better prognosis when they are positive for S118-P in these studies [32, 33]. These results are not easily reconciled with the previously mentioned studies (see [28, 31], Wigerup et al. unpublished data). Besides differences in patient series and tumor types, it is not clear which kinase activities in the tumors are resulting in S118 phosphorylation. In patients, both MAPK and RET expressions are associated with poor response to antihormonal therapy [53]. Activation of the ERK1/2 MAPK pathway apparently results in S118 phosphorylation, but it also induces a bypassing of the ER pathway, thereby rendering tumors hormone-independent. 

CDK7-mediated phosphorylation is indicative of an active ER*α*. Whereas the MAPK mechanism may well be responsible for a worse outcome of disease, irrespective of tamoxifen treatment, the CDK7 mechanism would indicate a proper functioning of ER*α*, being an adequate target for tamoxifen treatment [33].

### 3.3. S167

Serine 167 is phosphorylated by Akt, p90RSK, and mTOR/p70S6K. The latter kinase also phosphorylates S118. Akt is induced by EGF and IGF [35], p90RSK only by EGF stimulation [36]. EGFR overexpression induces S167 phosphorylation, increases binding of ER*α* to DNA, enhances the binding of coactivator SRC3 to ER*α* in the presence of E2, and consequently enhances transcription. Moreover, *in vitro,* S167-P reduces sensitivity to tamoxifen [25, 32]. Other kinases that target S167 include ERK1/2 MAPK [32, 37] and, upon E2 binding of ER*α*, casein kinase II (CK2) [38]. S167-P does not affect ligand binding [25].

The clinical data of S167 phosphorylation are conflicting. In ER*α*-positive, tamoxifen-treated patients, activated AKT (pAKT) is associated with high risk for relapse and decreased overall survival [39], which would imply that S167-P is associated with a worse disease outcome. However, it is important to realise that Akt, like ERK1/2 MAPK, has many other targets, which could well bypass the estrogen-receptor-dependent signaling. 

Notwithstanding, in a set of 75 primary breast carcinomas of patients with metastatic breast cancer who received first-line endocrine treatment after relapse, those staining high for S167-P relapsed later. The metastases responded well to endocrine treatment and S167-P correlated with longer survival after relapse. This implies that S167-P is a predictive marker for a good response to endocrine therapy [34, 37].

### 3.4. S282

Serine 282 resides in the hinge region and, like S167, can be phosphorylated by CK2. Estradiol increases phosphorylation of S282, stabilizes ER, and induces transcriptional activity [20]. In patients, low levels of S282 phosphorylation are associated with reduced overall survival in ER-positive breast tumors from tamoxifen-treated patients, suggesting that S282 phosphorylation can be predictive for response to tamoxifen [40].

### 3.5. S305

**Figure 2:**
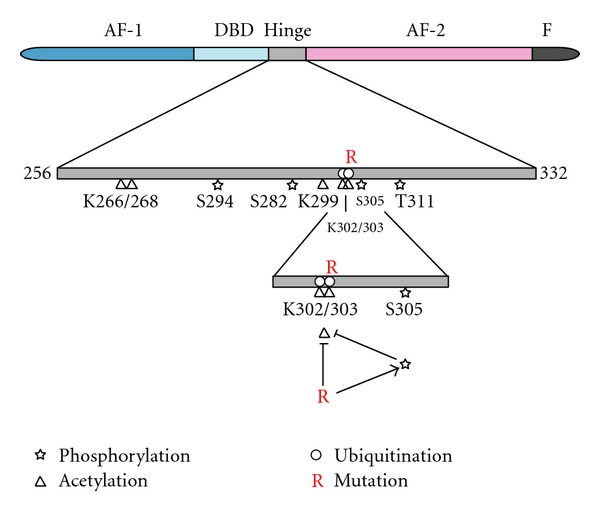
Posttranslational modifications in the ER*α* hinge region. S305 phosphorylation prevents acetylation of K302/303. The natural K303R mutation blocks K302/303 acetylation and stimulates S305 phosphorylation.

Serine 305 resides at the C-terminus of the hinge region that provides a centre of rotation to the total ER*α*. The region around Ser305 is a multifunctional domain that binds to many coregulatory proteins and is involved in the regulation of activity and stability of ER*α* [42]. Phosphorylation of Ser 305 occurs by protein kinase A and is associated with resistance to tamoxifen in patients (see [43–45], Wigerup et al. unpublished data). This domain also controls ER*α* ubiquitination and subsequent proteosomal degradation of ER*α*, that is influenced by ligands [46]. Different ligands can induce different conformations of ER*α* and hence affect accessibility to the hinge region for modifying proteins, such as ubiquitin ligases. This implies that ligands can be selective for specific posttranslational modifications. Within the hinge region, lysines K302/303 are involved in proteasomal degradation of ER*α* by fulvestrant and are the targets of polyubiquitination. K302 and K303 are both required for monoubiquitination by the BRCA1/BARD1 E3 ligase of E2- or tamoxifen-bound ER*α* [54]. K303 is also target for acetylation (inhibiting ER activity) and for methylation (stabilizing ER and increasing activity), whereas S305 phosphorylation prevents acetylation of K303 [55], thereby stimulating ER activity. The reverse is also true: a K303R mutation is frequently found in breast cancer, which prevents acetylation and increases phosphorylation of Ser305 by PKA [56]. These findings indicate that the hinge region is affected by various posttranslational modifications that affect structure and functioning of ER*α*. Some of these modifications and their cross-talk are shown in Figure 2.

Besides PKA, p21-associated kinase 1 (PAK1) has been suggested as an upstream kinase involved in the phosphorylation of Ser305. PAK1 phosphorylation of ER*α* S305 can lead to a secondary event on S118, presumably due to a conformational change of the estrogen receptor [15, 19]. PAK1 overexpression by itself is associated with resistance to tamoxifen *in vitro* [19] as well as in patients [32, 44, 45, 47]. Notably, in an experimental tamoxifen-resistant setting, tamoxifen induces PAK1, maintaining ER*α* in the tamoxifen-insensitive state [19]. The evidence that PAK1 phosphorylates S305 [19] was indirect and was not confirmed by a direct inspection of the phosphorylation of ER*α* Ser305 using specific antibodies or by the introduction of a dominant-active PAK1 into breast cancer cells [44]. Moreover, overexpression of PAK1 was not correlated with S305 phosphorylation in two different studies on breast cancer, indicating that these two events are independent (see [44], Wigerup et al. unpublished data). PKA phosphorylates S305, keeping ER in an active conformation when tamoxifen is bound, which means that it mimics an estrogen-bound ER [15]. This was not observed with overexpression of PAK1 [44]. 

Clinical studies show that tamoxifen resistance occurred in endocrine-treated patients with detectable S305-P in the primary human breast tumor [44]. Since S305 phosphorylation has no effect on patients that were not endocrine treated, this Ser305P markers appears to be a predictive marker for treatment outcome and not for general disease progression [43]. A combination of PAK-1, phosho-PKA, a marker of activated PKA, and the phosphorylated S305 marker identified approximately 60–70% of all tamoxifen resistant cases in breast cancer. This occurred in series of breast cancer from premenopausal and postmenopausal patients, in early to advanced stages of disease, indicating that the marker is independent of clinical stage of disease and of the hormonal status of the patient (see [31, 43, 45], Wigerup et al. unpublished data).

### 3.6. Y537

Tyrosine 537 is phosphorylated by the Src family tyrosine kinases. Phosphorylation of this tyrosine inhibits ER dimerisation and estrogen binding and reduces transcriptional activity of ER*α* [48]. Tyr 537 is located at the N-terminus of helix 12, and mutation of this Tyr into a nonphosphorylatable alanine facilitates the rotation of helix 12 into an active conformation of ER*α* in the absence of any ligand [49]. Phosphorylation by activated Src increases affinity for E2 and decreases affinity for tamoxifen [25]. Nonphosphorylatable mutants show ligand-independent transactivation, but this is inhibited by tamoxifen [32]. There is no apparent clinical evidence that Y537 phosphorylation influences tamoxifen response in patients. Of note, a naturally, but rarely occurring, Y537 mutation to asparagine (Y537N) in breast cancer metastasis constitutively activates the estrogen receptor by a conformational change of helix 12, which may contribute to breast cancer progression and resistance to endocrine treatment [50].

## 4. Other ER**α** Phosphorylation Sites with No (Known) Role in Tam Resistance

Several other phosphorylation sites of ER*α* have been found, which have not been associated with tamoxifen, either since tamoxifen was not included in the studies or because the phosphosite has not been included in clinical studies on tamoxifen resistance. These sites are briefly discussed below.

### 4.1. S46/47

Ser-46/47 phosphorylation plays a role in ligand-dependent activation of ER*α*. Mutation of Ser-46/47 or Ser-294 to alanine markedly reduced estradiol-dependent reporter activation. S47 phosphorylation may influence other posttranslational modifications of ER*α*. S46 is a putative recognition site for protein kinase C and seems to hold a predominant effect on transcriptional activity, rendering S47 phosphorylation a “bystander” effect [20].

### 4.2. Y52 and Y219

Tyrosine 52 and 219 are phosphorylated by c-Abl, a Src-like nonreceptor tyrosine kinase. Y219 phosphorylation affects ER dimerization and DNA binding. This results in enhanced ER*α* transcriptional activity, both in absence and presence of estradiol. Stabilisation of ER*α* through c-Abl ultimately leads to proliferation and invasion of breast tumor cells [21].

### 4.3. S154, S212, S294, and S554

Serine 154, 212, 294, and 554 are putative ER*α* phosphorylation sites discovered by mass spectrometry on phosphopeptides [20]. *In vitro*, an alanine mutation of S294 reduces estradiol-dependent transcription [20], suggesting that S294 phosphorylation is needed for a functional ER. Furthermore, S294 phosphorylation has been detected by immunohistochemistry (IHC) in human breast carcinoma but no significant effect of S294-P on tamoxifen response in terms of recurrence or overall survival has been observed [40]. The biological relevance of the other three serines remains to be tested.

### 4.4. S236

Serine 236 is located in the DNA binding domain (DBD). It is phosphorylated by PKA, upon which ER dimerisation and DNA binding in the absence of ligands are lost, rendering ER transcriptionally inactive [32], but both estradiol and tamoxifen can overcome this inhibition [17]. This would imply that S236-P in itself has no effect on tamoxifen sensitivity.

### 4.5. T311

Threonine 311 is the only known threonine phosphorylation site on ER*α*. An active RAS/MAPK pathway stimulates ER*α* phosphorylation at Thr-311 [21]. Phosphorylation of T311 can be detected by immunohistochemistry, but thus far has not been significantly associated with altered tamoxifen sensitivity in breast cancer patients [40].

### 4.6. S559

Serine 559, like Y537, resides in the F domain of the estrogen receptor, in helix 12. This is of particular interest, because the position of helix 12 determines interaction with coactivators and corepressors and regulates response to (ant)agonists. Therefore, S559 phosphorylation can probably influence ER binding to coregulators, such as SRC-1, by changing the position of helix 12 and as a consequence the response to ER ligands. S559 is targeted by CK2 [20]. Phosphorylation inhibits ligand-independent activation of ER*α*. ER*α* is phosphorylated at S559 in human breast carcinoma biopsies [41].

## 5. Main Points, Sideissues, and Interrelated Affairs

We presented, thus far, the effects of phosphorylation of relevant sites in ER*α* as single events. Of course, reality is more complex and modifications not only occur on ER*α* itself, but also take place on the associated cofactors and on targets outside the ER*α* signaling pathways that could have an effect on ER*α*-mediated signaling. Three examples below illustrate this point.

- Phosphorylation of CARM1, an arginine methyltransferase, by PKA [57]. Phosphorylation of CARM1 by PKA enhances its interaction with S448 in the LBD of ER*α* and creates a novel, more firm platform for binding of other cofactors. The net result is tamoxifen resistance by the buildup of a PKA-specific coactivator complex. Because the arginine methyltransferase CARM1 is involved in methylation of histones H3 and H4 that is crucial for transcription to occur, the ER*α*-phosphoCARM1 complex provides a specific regulatory unit for transcription. Still, additional events are needed for tamoxifen resistance, among which possibly the phosphorylation of ER*α* S305 by PKA.
- PAK1 phosphorylates an alternate, but in breast cancer frequently present, isoform of the SRC3 steroid-receptor cofactor (SRC3-3*δ*4), allowing it to bridge between EGF-R and FAK1 (focal adhesion kinase 1) and thereby activating ERK1/2 MAPK [58]. Activation of this pathway possibly renders breast tumor cells tamoxifen resistant. This provides a novel turn to the role of PAK1 overexpression in breast cancer.
- The selective activity of SRC-3 depends on specific phosphorylation of SRC3 [59]. SRC3 has six specific phosphorylation sites targeted by multiple kinases. These phosphorylated sites determine the optimal interaction with other transcription factors and are required for different physiological functions.

These three examples demonstrate that there is a complex interrelated network of regulatory circuits influencing ER transcriptional activity and that, by modification of one circuit, other circuits are affected. They also indicate that one particular mode of modification can have multiple effects. Most of the studies have addressed only one significant mode of action, but it is evident that many factors can play a role in the resistance to endocrine treatment.

## 6. Downstream Signaling/Gene Expression/Pathways

How does phosphorylation of ERa affect resistance to tamoxifen? The estrogen receptor is a nuclear receptor, which binds to specific sequences in the DNA and regulates the expression of ER-dependent genes. Phosphorylation of ER*α* can affect DNA binding, for example, by inhibiting dimerization of the receptor, and can influence ER*α* activity by changing the binding to coactivators or the orientation of components of the transcription factor complex. Which genes are then affected? In the classical way, an estradiol-bound estrogen receptor dimerizes, binds to an estrogen responsive element (ERE), and transcribes the gene that lies within its proximity. The estrogen receptor can also regulate transcription of genes in an indirect manner, by binding to other transcription factors: AP-1, SP-1 [60], or activated NFkB [61]. When these interactions occur, transcription of the AP-1-, SP-1-, or NFkB-dependent genes becomes also dependent on ER*α*. When tamoxifen is bound to ER*α*, the classical estrogen responsive genes are not expressed but tamoxifen-bound ER*α* has its own, different transcriptome, most likely generated through the nonclassical pathway [62, 63]. 

Different kinase pathways can be activated chemically in cells by adding growth factors (EGF or IGF) or cAMP, which induces PKA. This approach was used in a gene expression study on MCF7 breast cancer cells [64]. Kinases were activated, and gene expression profiles were compared in the presence or absence of tamoxifen. Tamoxifen treatment resulted in differential gene expression with either growth factor stimulation or PKA activation. Which of these genes is essential for tamoxifen resistance remains a crucial question. 

A more complete, but also more complex, picture arises from microarray analyses performed in tumors of tamoxifen-treated ER*α*-positive breast cancer patients. Frasor et al. described a set of genes associated with disease recurrence, a subset of which is associated with treatment with tamoxifen [63]. Loi et al. applied gene expression profiling in a similar way. They developed a gene classifier to predict clinical outcome in tamoxifen-treated ER*α*-positive breast cancer patients. This classifier contains genes involved in invasion (SLIT2 and RECK), anti-inflammatory response (TGFBR4, PTGER4, C3, and GNG2), and cell cycle regulation [65]. In later studies, this group validated a number of hits by qPCR and hence demonstrated that EZH2 downregulation is associated with a favourable outcome [66] and that downregulation of SIAH2, an E3-ubiquitin ligase, would imply tamoxifen resistance [67]. They also showed that an extracellular matrix cluster of genes (TIMP3, FN1, LOX, and SPARC) is associated with tamoxifen resistance [68]. In any of these studies, it is unclear whether phosphorylation of the estrogen receptor plays a role in tamoxifen resistance in these patients. Looking at multiple genes, instead of only one, could be more informative for treatment outcome. Therefore, Kok et al. compared three gene classifiers [69–71] for tamoxifen. This comparison indicates that a multigene approach would improve the prediction of response to tamoxifen [31]. 

There is as of yet only one microarray study on tamoxifen-treated ER*α*-positive human breast cancers that addresses a specific phosphorylation site, S305P, and the effect on gene expression. A pathway analysis highlighted several pathways being affected, including PKA, ERK1/2 MAPK, EGF signaling, CDK regulation, and interferon alpha signaling [44].

## 7. Discussion

In total, 19 phosphorylation sites have been identified in ER*α* thus far, as summarized in Figure 1. Phosphorylation of S167, S118, S282, and Y537 is beneficial for tamoxifen response according to experimental and, for S167, S118 and S282, because of reported clinical data. Tamoxifen resistance is likely to occur when S104/S106 or S305 is phosphorylated. The contribution of phosphorylation of other target sites to tamoxifen resistance remains to be determined. Some of these phosphorylation sites have been shown with FRET technology to induce a conformational change of ER*α*, when exposed to other antiestrogens, such as fulvestrant and raloxifene [6]. Thereby, they may affect the antagonistic behavior of these compounds but the molecular mechanisms remain to be elucidated.

Upstream of ER*α*, different kinase pathways are involved. Dependent on the pathway and the phosphorylation sites involved, tamoxifen response can be affected either directly through ER*α* modification or by activation of other signaling pathways. Phosphorylation of S118 is described as an example of this: an activated ERK1/2 MAPK pathway phosphorylates S118 but possibly induces tamoxifen resistance through the ERK1/2 MAPK pathway itself, rather than ER signaling. S118 phosphorylation by the ER-associated CDK7 indicates an activated ER which would imply a beneficial effect on tamoxifen treatment in patients. 

EGFR and cErbB2 can also affect ER signaling. Tamoxifen response may be restored by blocking EGFR with gefitinib [3, 72]. In a clinical study, blocking cErbB2 with trastuzumab restores ER*α* signaling in ER*α*-positive tumors and improves response to the aromatase inhibitor letrozole [73]. This would suggest a better response to tamoxifen as well. 

It is challenging to extrapolate experimental data from ER activation to the clinic and *vice versa*. In *in vitro *studies, it is feasible to examine differential gene expression after treatment and compare the profile before and after treatment, or in absence or presence of phosphorylation. Translation of this information to the clinic can, however, be troublesome, since adjuvant tamoxifen treatment is started after surgical removal of the primary tumor. The *in vitro* experiments measure gene expression changes associated with acquired resistance, whereas investigation of primary tumors that respond better to treatment highlights genes which play a role in intrinsic resistance to tamoxifen. Because primary tumors have not been exposed to tamoxifen, endocrine treatment cannot be a selective factor for these resistance markers. They may occur at random during normal tumor development or may well coincide with other tumor progression markers. For example, overexpression of cErbB2 or of EGFR in breast cancer marks worse course of disease not only in ER*α*-negative, but also in ER*α*-positive tumors and is a marker for tamoxifen resistance as well [74]. 

Phosphorylation of Ser305 is a marker for intrinsic resistance to tamoxifen. It is not associated with disease progression in the absence of tamoxifen treatment [44]. It is also a marker for the choice of treatment, since a combination of S305-P, S118-P, and overexpression of SRC-1 or cyclin D1 coactivators dictates resistance to different antiestrogens [14, 15]. Since outgrowth of micrometastases into tamoxifen resistant tumors occurs over longer periods of time (up to 15 years), extra alterations in the micrometastases outgrowths, in addition to the S305 phosphorylation status, potentially influence tamoxifen resistance. Phosphorylation of Ser305, however, was still maintained in the few metastases samples that could be examined [44]. Alternatively, one could study acquired and intrinsic resistance during neoadjuvant treatment with antiestrogens, where patients are treated up to three months prior to the surgical removal of the primary tumor. Hence, samples can be obtained before and after treatment for comparison [75]. In another study by this group, activation of the ERK1/2/MAPK pathway was a major factor associated with acquired resistance to tamoxifen [74].

Phosphorylation of S305 has experimentally been linked to resistance to tamoxifen, because of an altered conformation of ER, where tamoxifen behaves as an agonist in FRET and expression reporter assays [15]. In patients, S305P was associated with alterations in the PKA pathway that result in stimulation of PKA activity [15, 44]. Also experimental enhancement of PKA activity in breast tumor cells led to proliferation of T47D breast tumor cells in the presence of tamoxifen [15]. It is, however, still possible that S305P is a marker for tamoxifen resistance without any direct involvement. It could merely mark PKA related events that bypass the estrogen receptor and hence induce tamoxifen resistance. The altered orientation of components of the transcription factor complex and the conformational changes in ER*α* strongly suggest, but are no proof of, a direct involvement. S305-P is, however, one of the few selective markers that predict resistance to tamoxifen in breast cancer patients. Definition of the activation of relevant signaling pathways in the ER*α*-positive breast tumors (that constitute the bulk of human breast cancers) prior to endocrine treatment is essential for treatment success and will ultimately lead to personalised treatment of breast cancer patients.

## Figures and Tables

**Table 1:** Putative ER*α* phosphosites, the kinases that target them, and the effect on tamoxifen response.

Phosphosite	Kinases	Tamoxifen	Reference
S46/47	PKC		[20]
Y52	c-Abl		[21]
S102/4/6	GSK-3, ERK1/2 MAPK, CDK2	Resistance	[20, 22–24]
S118	CDK2, ERK1/2 MAPK, RAS/MAPK, GSK-3, CDK7, IKK*α*, mTOR/p70S6K	Dual effect	[18, 25–34]
S154			[20]
S167	ERK1/2 MAPK, p90RSK, CK2, Akt, mTOR/p70S6K	Sensitivity	[25, 30, 32, 34–39]
S212			[20]
Y219	c-Abl		[21]
S236	PKA		[17, 32]
S282	CK2	Sensitivity	[20, 40, 41]
S294			[20, 40]
S305	PKA, PAK1?	Resistance	[15, 19, 32, 42–47]
T311	RAS/MAPK		[21, 40]
Y537	Src Y kinases	Dual effect	[25, 32, 48–50]
S554			[20]
S559	CK2		[20, 41]

## References

[1] Ali S, Coombes RC (2002). Endocrine-responsive breast cancer and strategies for combating resistance. *Nature Reviews Cancer*.

[2] Musgrove EA, Sutherland RL (2009). Biological determinants of endocrine resistance in breast cancer. *Nature Reviews Cancer*.

[3] Shou J, Massarweh S, Osborne CK (2004). Mechanisms of tamoxifen resistance: increased estrogen receptor-HER2/neu cross-talk in ER/HER2-positive breast cancer. *Journal of the National Cancer Institute*.

[4] Massarweh S, Osborne CK, Creighton CJ (2008). Tamoxifen resistance in breast tumors is driven by growth factor receptor signaling with repression of classic estrogen receptor genomic function. *Cancer Research*.

[5] Osborne CK, Coronado-Heinsohn EB, Hilsenbeck SG (1995). Comparison of the effects of a pure steroidal antiestrogen with those of tamoxifen in a model of human breast cancer. *Journal of the National Cancer Institute*.

[6] Zwart W, Griekspoor A, Rondaij M, Verwoerd D, Neefjes J, Michalides R (2007). Classification of anti-estrogens according to intramolecular FRET effects on phospho-mutants of estrogen receptor *α*. *Molecular Cancer Therapeutics*.

[7] Zwart W, de Leeuw R, Rondaij M, Neefjes J, Mancini MA, Michalides R (2010). The hinge region of the human estrogen receptor determines functional synergy between AF-1 and AF-2 in the quantitative response to estradiol and tamoxifen. *Journal of Cell Science*.

[8] Ruff M, Gangloff M, Wurtz JM, Moras D (2000). Structure-function relationships in DNA- and ligand-binding domains of estrogen receptors. *Breast Cancer Research*.

[9] Herynk MH, Fuqua SAW (2004). Estrogen receptor mutations in human disease. *Endocrine Reviews*.

[10] Heery DM, Kalkhoven E, Hoare S, Parker MG (1997). A signature motif in transcriptional co-activators mediates binding to nuclear receptors. *Nature*.

[11] Shiau AK, Barstad D, Radek JT (2002). Structural characterization of a subtype-selective ligand reveals a novel mode of estrogen receptor antagonism. *Nature Structural Biology*.

[12] Shiau AK, Barstad D, Loria PM (1998). The structural basis of estrogen receptor/coactivator recognition and the antagonism of this interaction by tamoxifen. *Cell*.

[13] Nagy L, Schwabe JWR (2004). Mechanism of the nuclear receptor molecular switch. *Trends in Biochemical Sciences*.

[14] Zwart W, Griekspoor A, Berno V (2007). PKA-induced resistance to tamoxifen is associated with an altered orientation of ER*α* towards co-activator SRC-1. *The EMBO Journal*.

[15] Michalides R, Griekspoor A, Balkenende A (2004). Tamoxifen resistance by a conformational arrest of the estrogen receptor *α* after PKA activation in breast cancer. *Cancer Cell*.

[16] Zwart W, Rondaij M, Jalink K (2009). Resistance to antiestrogen arzoxifene is mediated by overexpression of cyclin D1. *Molecular Endocrinology*.

[17] Chen D, Pace PE, Coombes RC, Ali S (1999). Phosphorylation of human estrogen receptor *α* by protein kinase A regulates dimerization. *Molecular and Cellular Biology*.

[18] Kato S, Endoh H, Masuhiro Y (1995). Activation of the estrogen receptor through phosphorylation by mitogen-activated protein kinase. *Science*.

[19] Rayala SK, Talukder AH, Balasenthil S (2006). P21-activated kinase 1 regulation of estrogen receptor-*α* activation involves serine 305 activation linked with serine 118 phosphorylation. *Cancer Research*.

[20] Williams CC, Basu A, El-Gharbawy A, Carrier LM, Smith CL, Rowan BG (2009). Identification of four novel phosphorylation sites in estrogen receptor *α*: impact on receptor-dependent gene expression and phosphorylation by protein kinase CK2. *BMC Biochemistry*.

[21] He X, Zheng Z, Song T (2010). C-Abl regulates estrogen receptor *α* transcription activity through its stabilization by phosphorylation. *Oncogene*.

[22] Thomas RS, Sarwar N, Phoenix F, Coombes RC, Ali S (2008). Phosphorylation at serines 104 and 106 by Erk1/2 MAPK is important for estrogen receptor-*α* activity. *Journal of Molecular Endocrinology*.

[23] Chen D, Washbrook E, Sarwar N (2002). Phosphorylation of human estrogen receptor *α* at serine 118 by two distinct signal transduction pathways revealed by phosphorylation-specific antisera. *Oncogene*.

[24] Rogatsky I, Trowbridge JM, Garabedian MJ (1999). Potentiation of human estrogen receptor *α* transcriptional activation through phosphorylation of serines 104 and 106 by the cyclin A-CDK2 complex. *Journal of Biological Chemistry*.

[25] Likhite VS, Stossi F, Kim K, Katzenellenbogen BS, Katzenellenbogen JA (2006). Kinase-specific phosphorylation of the estrogen receptor changes receptor interactions with ligand, deoxyribonucleic acid, and coregulators associated with alterations in estrogen and tamoxifen activity. *Molecular Endocrinology*.

[26] Vendrell JA, Bieche I, Desmeiz C (2005). Molecular changes associated with the agonist activity of hydroxy-tamoxifen and the hyper-response to estradiol in hydroxy-tamoxifen-resistant breast cancer cell lines. *Endocrine-Related Cancer*.

[27] Cheng J, Zhang C, Shapiro DJ (2007). A functional serine 118 phosphorylation site in estrogen receptor-*α* is required for down-regulation of gene expression by 17*β*-estradiol and 4-hydroxytamoxifen. *Endocrinology*.

[28] Weitsman GE, Li L, Skliris GP (2006). Estrogen receptor-*α* phosphorylated at Ser118 is present at the promoters of estrogen-regulated genes and is not altered due to HER-2 overexpression. *Cancer Research*.

[29] Chen D, Riedl T, Washbrook E (2000). Activation of estrogen receptor *α* by S118 phosphorylation involves a ligand-dependent interaction with TFIIH and participation of CDK7. *Molecular Cell*.

[30] Morandi A, Plaza-Menacho I, Isacke CM (2011). RET in breast cancer: functional and therapeutic implications. *Trends in Molecular Medicine*.

[31] Kok M, Holm-Wigerup C, Hauptmann M (2009). Estrogen receptor-*α* phosphorylation at serine-118 and tamoxifen response in breast cancer. *Journal of the National Cancer Institute*.

[32] Riggins RB, Schrecengost RS, Guerrero MS, Bouton AH (2007). Pathways to tamoxifen resistance. *Cancer Letters*.

[33] Sarwar N, Kim JS, Jiang J (2006). Phosphorylation of ER*α* at serine 118 in primary breast cancer and in tamoxifen-resistant tumours is indicative of a complex role for ER*α* phosphorylation in breast cancer progression. *Endocrine-Related Cancer*.

[34] Yamashita H, Nishio M, Toyama T (2008). Low phosphorylation of estrogen receptor *α* (ER*α*) serine 118 and high phosphorylation of ER*α* serine 167 improve survival in ER-positive breast cancer. *Endocrine-Related Cancer*.

[35] Martin MB, Franke TF, Stoica GE (2000). A role for Akt in mediating the estrogenic functions of epidermal growth factor and insulin-like growth factor I. *Endocrinology*.

[36] Joel PB, Smith J, Sturgill TW, Fisher TL, Blenis J, Lannigan DA (1998). pp90^rsk1^ regulates estrogen receptor-mediated transcription through phosphorylation of Ser-167. *Molecular and Cellular Biology*.

[37] Yamashita H, Nishio M, Kobayashi S (2005). Phosphorylation of estrogen receptor alpha serine 167 is predictive of response to endocrine therapy and increases postrelapse survival in metastatic breast cancer. *Breast Cancer Research*.

[38] Arnold SF, Obourn JD, Jaffe H, Notides AC (1994). Serine 167 is the major estradiol-induced phosphorylation site on the human estrogen receptor. *Molecular Endocrinology*.

[39] Kirkegaard T, Witton CJ, McGlynn LM (2005). AKT activation predicts outcome in breast cancer patients treated with tamoxifen. *Journal of Pathology*.

[40] Skliris GP, Nugent ZJ, Rowan BG, Penner CR, Watson PH, Murphy LC (2010). A phosphorylation code for oestrogen receptor-*α* predicts clinical outcome to endocrine therapy in breast cancer. *Endocrine-Related Cancer*.

[41] Skliris GP, Rowan BG, Al-Dhaheri M (2009). Immunohistochemical validation of multiple phospho-specific epitopes for estrogen receptor *α* (ER*α*) in tissue microarrays of ER*α* positive human breast carcinomas. *Breast Cancer Research and Treatment*.

[42] Barone I, Brusco L, Fuqua SAW (2010). Estrogen receptor mutations and changes in downstream gene expression and signaling. *Clinical Cancer Research*.

[43] Holm C, Kok M, Michalides R (2009). Phosphorylation of the oestrogen receptor *α* at serine 305 and prediction of tamoxifen resistance in breast cancer. *Journal of Pathology*.

[44] Kok M, Zwart W, Holm C (2011). PKA-induced phosphorylation of ER*α* at serine 305 and high PAK1 levels is associated with sensitivity to tamoxifen in ER-positive breast cancer. *Breast Cancer Research and Treatment*.

[45] Bostner J, Skoog L, Fornander T, Nordenskjöld B, Stål O (2010). Estrogen receptor-*α* phosphorylation at serine 305, nuclear p21-activated kinase 1 expression, and response to tamoxifen in postmenopausal breast cancer. *Clinical Cancer Research*.

[46] Wijayaratne AL, McDonnell DP (2001). The human estrogen receptor-*α* is a ubiquitinated protein whose stability is affected differentially by agonists, antagonists, and selective estrogen receptor modulators. *Journal of Biological Chemistry*.

[47] Bostner J, Ahnström Waltersson M, Fornander T, Skoog L, Nordenskjöld B, Stål O (2007). Amplification of CCND1 and PAK1 as predictors of recurrence and tamoxifen resistance in postmenopausal breast cancer. *Oncogene*.

[48] Arnold SF, Melamed M, Vorojeikina DP, Notides AC, Sasson S (1997). Estradiol-binding mechanism and binding capacity of the human estrogen receptor is regulated by tyrosine phosphorylation. *Molecular Endocrinology*.

[49] Skafar DF (2000). Formation of a powerful capping motif corresponding to start of "Helix 12" in agonist-bound estrogen receptor-*α* contributes to increased constitutive activity of the protein. *Cell Biochemistry and Biophysics*.

[50] Zhang QX, Borg A, Wolf DM, Oesterreich S, Fuqua SAW (1997). An estrogen receptor mutant with strong hormone-independent activity from a metastatic breast cancer. *Cancer Research*.

[51] Michalides R, van Tinteren H, Balkenende A (2002). Cyclin A is a prognostic indicator in early stage breast cancer with and without tamoxifen treatment. *British Journal of Cancer*.

[52] Duplessis TT, Williams CC, Hill SM, Rowan BG (2011). Phosphorylation of estrogen receptor *α* at serine 118 directs recruitment of promoter complexes and gene-specific transcription. *Endocrinology*.

[53] Gee JMW, Robertson JFR, Ellis IO, Nicholson RI (2001). Phosphorylation of ERK1/2 mitogen-activated protein kinase is associated with poor response to anti-hormonal therapy and decreased patient survival in clinical breast cancer. *International Journal of Cancer*.

[54] Eakin CM, MacCoss MJ, Finney GL, Klevit RE (2007). Estrogen receptor *α* is a putative substrate for the BRCA1 ubiquitin ligase. *Proceedings of the National Academy of Sciences of the United States of America*.

[55] Cui Y, Zhang M, Pestell R, Curran EM, Welshons WV, Fuqua SAW (2004). Phosphorylation of estrogen receptor *α* blocks its acetylation and regulates estrogen sensitivity. *Cancer Research*.

[56] Giordano C, Cui Y, Barone I (2010). Growth factor-induced resistance to tamoxifen is associated with a mutation of estrogen receptor *α* and its phosphorylation at serine 305. *Breast Cancer Research and Treatment*.

[57] Carascossa S, Dudek P, Cenni B, Briand PA, Picard D (2010). CARM1 mediates the ligand-independent and tamoxifen-resistant activation of the estrogen receptor *α* by cAMP. *Genes and Development*.

[58] Long W, Yi P, Amazit L (2010). SRC-3Δ4 mediates the interaction of EGFR with FAK to promote cell migration. *Molecular Cell*.

[59] Wu RC, Qin J, Yi P (2004). Selective phosphorylations of the SRC-3/AIB1 coactivator integrate genomic reponses to multiple cellular signaling pathways. *Molecular Cell*.

[60] Gustafsson JÅ (2000). An update on estrogen receptors. *Seminars in Perinatology*.

[61] Zhou Y, Eppenberger-Castori S, Eppenberger U, Benz CC (2005). The NF*κ*B pathway and endocrine-resistant breast cancer. *Endocrine-Related Cancer*.

[62] Gadal F, Starzec A, Bozic C (2005). Integrative analysis of gene expression patterns predicts specific modulations of defined cell functions by estrogen and tamoxifen in MCF7 breast cancer cells. *Journal of Molecular Endocrinology*.

[63] Frasor J, Chang EC, Komm B (2006). Gene expression preferentially regulated by tamoxifen in breast cancer cells and correlations with clinical outcome. *Cancer Research*.

[64] Dudek P, Picard D (2008). Genomics of signaling crosstalk of estrogen receptor *α* in breast cancer cells. *PLoS One*.

[65] Loi S, Haibe-Kains B, Desmedt C (2008). Predicting prognosis using molecular profiling in estrogen receptor-positive breast cancer treated with tamoxifen. *BMC Genomics*.

[66] Reijm EA, Jansen MPHM, Ruigrok-Ritstier K (2011). Decreased expression of EZH2 is associated with upregulation of ER and favorable outcome to tamoxifen in advanced breast cancer. *Breast Cancer Research and Treatment*.

[67] Jansen MPHM, Ruigrok-Ritstier K, Dorssers LCJ (2009). Downregulation of SIAH2, an ubiquitin E3 ligase, is associated with resistance to endocrine therapy in breast cancer. *Breast Cancer Research and Treatment*.

[68] Helleman J, Jansen MPHM, Ruigrok-Ritstier K (2008). Association of an extracellular matrix gene cluster with breast cancer prognosis and endocrine therapy response. *Clinical Cancer Research*.

[69] Jansen MPHM, Foekens JA, van Staveren IL (2005). Molecular classification of tamoxifen-resistant breast carcinomas by gene expression profiling. *Journal of Clinical Oncology*.

[70] Paik S, Shak S, Tang G (2004). A multigene assay to predict recurrence of tamoxifen-treated, node-negative breast cancer. *The New England Journal of Medicine*.

[71] Jansen MPHM, Sieuwerts AM, Look MP (2007). HOXB13-to-IL17BR expression ratio is related with tumor aggressiveness and response to tamoxifen of recurrent breast cancer: a retrospective study. *Journal of Clinical Oncology*.

[72] Creighton CJ, Massarweh S, Huang S (2008). Development of resistance to targeted therapies transforms the clinically associated molecular profile subtype of breast tumor xenografts. *Cancer Research*.

[73] Sabnis G, Schayowitz A, Goloubeva O, Macedo L, Brodie A (2009). Trastuzumab reverses letrozole resistance and amplifies the sensitivity of breast cancer cells to estrogen. *Cancer Research*.

[74] Gutierrez MC, Detre S, Johnston S (2005). Molecular changes in tamoxifen-resistant breast cancer: relationship between estrogen receptor, HER-2, and p38 mitogen-activated protein kinase. *Journal of Clinical Oncology*.

[75] Ellis MJ, Tao Y, Luo J (2008). Outcome prediction for estrogen receptor-positive breast cancer based on postneoadjuvant endocrine therapy tumor characteristics. *Journal of the National Cancer Institute*.

